# Evaluation of Different Adiposity Indices and Association with Metabolic Syndrome Risk in Obese Children: Is there a Winner?

**DOI:** 10.3390/ijms21114083

**Published:** 2020-06-08

**Authors:** Alessandro Leone, Sara Vizzuso, Paolo Brambilla, Chiara Mameli, Simone Ravella, Ramona De Amicis, Alberto Battezzati, Gianvincenzo Zuccotti, Simona Bertoli, Elvira Verduci

**Affiliations:** 1 International Center for the Assessment of Nutritional Status (ICANS), Department of Food, Environmental and Nutritional Sciences (DeFENS), University of Milan, Via Sandro Botticelli 21, 20133 Milan, Italy; alessandro.leone1@unimi.it (A.L.); simone.ravella@unimi.it (S.R.); ramona.deamicis@unimi.it (R.D.A.); alberto.battezzati@unimi.it (A.B.); 2 Department of Health Sciences, University of Milan, 20133 Milan, Italy; sara.vizzuso@unimi.it (S.V.); elvira.verduci@unimi.it (E.V.); 3 Family Pediatrician, ATS Città Metropolitana Milano, 20122 Milan, Italy; paolo.brambilla3@gmail.com; 4 Department of Pediatrics Vittore Buzzi Children’s Hospital, University of Milan, 20154 Milan, Italy; chiara.mameli@unimi.it (C.M.); gianvincenzo.zuccotti@unimi.it (G.Z.); 5 Istituto Auxologico Italiano, IRCCS, Lab of Nutrition and Obesity Research, 20145 Milan, Italy

**Keywords:** adiposity indices, metabolic syndrome, childhood obesity

## Abstract

Body shape index (ABSI) and triponderal mass index (TMI) have been recently associated with cardiovascular risk in adults. A cross-sectional study was conducted to evaluate the relationship between different anthropometric adiposity indexes and metabolic syndrome (MetS) in Caucasian obese children and adolescents. Consecutive obese children aged ≥7 years have been enrolled. Anthropometric parameters, body composition (by bioelectrical impedance), and systolic and diastolic blood pressure have been measured. Fasting blood samples have been analyzed for lipids, insulin, glucose. A multivariate logistic regression analyses, with body mass index *z*-score, waist to height ratio, ABSI *z*-score, TMI, conicity index as predictors for MetS (IDEFICS and IDF criteria according to age) has been performed. Four hundred and three (179 boys and 224 girls) obese children, aged 7–20 years, have been evaluated. When we explored the joint contribution of each anthropometric and adiposity index of interest and BMIz on the risk of MetS, we found that the inclusion of ABSIz improved the prediction of MetS compared to BMIz alone. ABSI-BMI can be a useful index for evaluating the relative contribution of central obesity to cardiometabolic risk in clinical management of obese children and adolescents.

## 1. Introduction

Childhood obesity is considered one of the most serious global public health challenges in the 21st century [1] in terms of prevalence and economic burden [2,3]. According to WHO data, 41 million children under the age of 5 years and 340 million children and adolescents aged 5–19 years are overweight or obese worldwide [4]. The latest WHO European Childhood Obesity Surveillance Initiative (COSI) data collection showed an increase in obesity prevalence in school-aged children (6–9 years) now affecting 21% of boys and 19% of girls [5].

Obesity may be associated with adverse health effects during childhood and with an increased risk of metabolic and cardiovascular morbidity and mortality later in life. Moreover, obese children can develop dyslipidemia, hypertension, and disorders of glucose metabolism, hallmarks of metabolic syndrome (MetS), more frequently during adolescence [6,7]. These long-term effects, especially if obesity status develops early in life, seem to be related to imbalanced gut microbiome, inflammation, impaired insulin signaling, and metabolic dysregulation [8]. Intensive lifestyle modifications, involving diet, physical activity, and behavioral changes are the key points of the actual guidelines to prevent and manage childhood obesity [9,10,11,12]. A reference method for the assessment of body composition is dual-energy X-ray absorptiometry (DXA). However, DXA, as well as air displacement plethysmography, is not widely available (partly because of the associated costs) as anthropometry, a simple, noninvasive, and inexpensive technique.

Body mass index (BMI) is the most used indicator in epidemiological studies and clinical setting and it is used as surrogate for the evaluation of body composition [13] but is not able to distinguish fat from lean mass, nor it is indicative of the adipose tissue distribution [14]. 

In children, the use of age- and gender-adjusted BMI *z*-score has been recommended instead of BMI; however, the association of childhood BMI *z*-score with cardiometabolic risk is nonlinear [15]. 

Different adiposity indexes have been evaluated to identify those with the best predictiveness for MetS. Waist circumference (WC) has been investigated in evaluation of body composition and cardiometabolic risk, as WC reflects also fat distribution and fat percentage [16]. WC has been shown to be a better predictor of hypertension and impaired glucose metabolism in adolescents when compared to BMI [17,18]. Perona suggested that WC is one of the strongest anthropometric discriminator of MetS among Spanish adolescents [19].

Waist to height ratio (WHtR) has been raised as a good marker of MetS in childhood [20] and it could have greater practical advantages over BMI and WC alone [21]. Joyce has suggested WHtR as a useful screening measure to identify adolescents at higher risk of hypertension in routine primary-level health services [22]. Nevertheless, some studies were not able to demonstrate a significant difference in the predictive abilities of BMI, WC, WHtR [23,24,25]. Recently, a new index, the body shape index (ABSI), related to the abdominal to peripheral fat ratio, has been specifically developed to stress the importance of waist circumference in abdominal obesity, associated with metabolic and cardiovascular alterations [26,27]. Since the ABSI is calculated using both waist circumference and BMI, it is possible that it may be a better predictor of BMI in assessing disease risk [27]. In adults, ABSI is a well-demonstrated predictor of total mortality and of incident cardiovascular disease (CVD) [28] with an accuracy similar to that of common laboratory measurements [29]. Concerning cardio-metabolic risk factor, we recently demonstrated that it is a useful index for evaluating the independent contribution of WC, in addition to that of BMI, as a surrogate for central obesity by ultrasonography [26]. Indeed, ABSI has been shown to be significantly associated with cardiometabolic risk markers in a pediatric overweight or obese population [30]. Also triponderal mass index (TMI) has been recently suggested as useful tools in the evaluation of body composition [31] and have been studied as predictors of MetS [32] in children and adolescents. It is still controversial whether conicity index (C-Index) could be useful to screen for MetS [33].

The aim of this study is to evaluate the relationship between different anthropometric adiposity indexes (AAIs) and metabolic syndrome (MetS) and to identify which of the AAIs allows a better assessment of the probability of having MetS in Caucasian obese children and adolescents aged ≥7 years.

## 2. Results

Table 1 shows the anthropometric measurements, the adiposity indexes and the biochemical parameters of the recruited children and adolescents.

Table 2 shows the distribution of MetS and its components in the total sample and according to the age class. Overall, MetS was detected in 19.9% of subjects.

Table 3 shows the regression coefficients, pseudo-R2 and AIC values for the logistic regression models used to investigate the association of the anthropometric and adiposity indices of interest (BMIz, ABSIz, TMI, C-Index, and WHtR) with the risk of MetS according to the age group.

In children aged < 10 years, we found that only BMIz was associated with the risk of MetS. In older children and in adolescents, all anthropometric and adiposity indices of interest, except ABSIz, were associated with MetS risk. In this age group, BMIz and WHtR had the lowest AIC value, suggesting an equal ability to predict MetS.

When we stratified the analysis for sex, we found C-Index and WHtR both associated with the MetS risk in female aged ≥10 years, with C-Index having a slightly better ability to predict MetS compared to WHtR (AIC = 127 vs. 128). In males aged ≥10 years, all anthropometric and adiposity indices, with the only exception for ABSIz, were associated with the risk of MetS, and BMIz had the best predictive ability (AIC = 107).

We also explored the association of anthropometric and adiposity indices with individual components of MetS (Supplementary Table S1). In children aged < 10 years, BMIz was associated with higher risk of high blood pressure. BMIz was also the best predictor of high blood pressure and low HDL in females and males aged ≥ 10 years, respectively. In females aged ≥ 10 years, C-Index was the best predictor of high triglycerides. In males ≥ 10 years, TMI better predicted high blood pressure, while WHtR better predicted high triglycerides.

When we explored the joint contribution of each anthropometric and adiposity index of interest and BMIz on the risk of metabolic syndrome, we found that the inclusion of ABSIz improved the prediction of MetS compared to BMIz alone in children aged ≥ 10 years (Table 4). Because of evident collinearity problems, we could not investigate the joint contribution of other indices and BMI in the prediction of MetS.

## 3. Discussion

In the present study, we tested for the first time the relationship between different anthropometric and adiposity indexes and metabolic syndrome (MetS) risk in a large sample of Caucasian obese children and adolescents taking into account the effects of sex and age.

We found that BMIz was independently associated and was associated with a better estimate of the probability of having MetS, compared to other indices. Moreover, the joint use of BMIz and ABSIz was associated with a better estimate of the probability of having MetS as compared to BMIz or ABSIz alone and to other indices. These findings did not change when we restricted the analysis to children aged over 10 years. Interestingly, when we stratified the analysis for sex, in subjects aged ≥10 years, we found that only C-Index and WHtR were both associated with the MetS risk in female, whereas in males all anthropometric and adiposity indices, with the only exception for ABSIz, were associated with the risk of MetS and BMIz suggesting that a gender difference in waist circumference effect on MetS is relevant.

Our results are interesting considering that ABSI, which express the WC relative to height and weight, has been recently proposed as a new method to better evaluate the cardiometabolic risk compared to BMI alone, both in children and in adults [26,30,31]. In a similar work, but conducted just among adolescents, WC and abdominal volume index have been the strongest anthropometric discriminator of MetS [19]. It has to be noted that in Perona study [19] the prevalence of MetS was 7% and 6.1% for 13.2 (1.2) years boys and girls, respectively, using IDF criteria. In our study, the prevalence of MetS was 11.4% considering 10–16 years old participants, then about two times greater than the Spanish study [19], probably because of higher mean value of BMI of participants in our study.

MetS is a complex disorder defined by a cluster of nutritional and biochemical factors that directly increase the risk of cardiovascular diseases and type 2 diabetes and its increasing prevalence in both childhood and young adulthood has future implications to the global health burden. Our results on the association of anthropometric and adiposity indices highlighted their different role as predictor of risk for individual components of MetS according to sex and age. The age of obesity onset could reflect a different cardiometabolic risk. Indeed, it was reported that patients who were obese at age ≥ 20 years had significantly higher odds of having T2D than those with the onset of obesity before 20 years [34]. Indeed it should be noted that patients with a younger age at obesity onset are less likely to clinically manifest consequences of obesity, such as diabetes or hypertension, compared to patients with adult onset of obesity [35].

However, another recent study conducted in a cohort of young women, aged 18–23 years, underlined the importance of timing of obesity in the development of T2D, suggesting that preventing the onset of obesity may substantially reduce the risk of developing diabetes [36]. Pacheco et al. have noticed in a cohort of 673 Chilean patients assessed both at 5 years and in adolescent age, that, in a multivariable model, an early onset of obesity independently contributed to a higher MetS risk score in adolescence [37].

Unfortunately, there is still no universally accepted and clearly defined diagnostic criteria for MetS, especially in childhood. Indeed, diagnostic criteria are different in children, adolescents, and young adulthood, and there is no way to use unified criteria, at present. Nonetheless, in agreement with the previous by Kassi et al. [38] in adults, our results even more stringently underscore the need for clear MetS criteria in childhood, because of the existing controversies in this field and the need to expand knowledge on the childhood aspect of the MetS.

Several studies demonstrated that visceral abdominal fat tissue (VAT) plays a central role in the pathogenesis of MetS both in adults and in children [39]. Computed tomography (CT) and magnetic resonance imaging (MRI) are the reference methods for the assessment of VAT but they cannot be used in routine clinical practice and epidemiological research. However, ultrasonography and bioelectrical impedance analysis has been a validated, cheap, and noninvasive alternative to reference methods [9,40]. ABSI is positively correlated with visceral adiposity and has been also shown to be positively associated with visceral fat thickness and area estimated respectively by ultrasonography and bioelectrical impedance analysis [41,42]. VAT by CT and MRI correlation with ABSI has never been investigated.

The role of abdominal adiposity as risk factor for cardiovascular and metabolic obesity-related alterations has been extensively studied in adult population, but in children has not yet been fully elucidated [43]. Visceral obesity may partly be a marker of a dysmetabolic state and partly a cause of the metabolic syndrome. A recent systematic review showed that abdominal fat deposition in children and adolescents increase the risk of cardio-metabolic alterations [44]. However, controversies exist on the definition of abdominal obesity in the pediatric age group. BMI itself cannot differentiate between fat and fat-free mass and does not always relate to central obesity. In addition, the recent increase in mean BMI of children and adolescents has been accompanied by an even steeper increase in WC [44]. Although WC is a better marker of abdominal fat accumulation than the BMI, an elevated waistline alone is not sufficient to diagnose visceral obesity and therefore the MetS risk. Indeed by using WC alone for distinguishing between subcutaneous and visceral fat mass is not possible [45]. It is for this reason that new adiposity indexes have been recently studied, among these ABSI, that normalizing the WC to height and weight could be better related to the abdominal to peripheral fat ratio.

The present study has some strengths and limitations. Among the strengths, we would like to highlight the large sample size with a large range of age of both sex, which contributes to obtaining robust results that will be useful in future comparable studies. Additionally, the studied sample can be considered a homogeneous sample as participating children and adolescents belonged to the same geographical region, with supposed similar culture, lifestyle, and eating habits.

Among the limitations of the study, we must include a self-selected sample of Caucasian children and adolescent. Our findings are not necessarily applicable to general populations and to other ethnic groups Therefore, more studies are needed to determine whether the results obtained are consistent using large sample of same age children.

Moreover, it is to be noted that while for BMI *z*-score and WHtR there are cut-off values associated with adiposity [46,47] and cardiometabolic risk [48], they do not exist for the other adiposity indexes analyzed in the study. A higher ABSI may correspond to a larger fraction of visceral fat [27] and since the ABSI is calculated using both waist circumference and BMI, it is possible that it may be a better predictor of BMI in assessing disease risk [27] but from the analysis of our data, in consideration of the sample size, it was not possible to identify specific cut-off for ABSI to define the obesity, central obesity, and consequently the cardiometabolic risk in these patients. Further studies are needed to evaluate this specific topic, including also the evaluation of VAT.

In conclusion, the joint use of ABSI and BMI allows a better assessment of the probability of METs compared to BMI alone and to the other AAIs tested. ABSI-BMI can be a useful index, as opposed to WC, for evaluating the relative contribution of central obesity to cardiometabolic risk in the clinical management of obese children and adolescents. Further studies aiming to evaluate the capability of ABSI-BMI to jointly predict longitudinal outcomes in pediatric population are warranted.

## 4. Materials and Methods

### 4.1. Study Population

We carried out a cross-sectional study on 403 Caucasian obese children and adolescents recruited at International Center for the Assessment of Nutritional Status (ICANS), University of Milan (59.6%) and San Paolo Hospital (40.4%), Department of Health Sciences, University of Milan, between January 2009 and June 2018. Inclusion criteria of the study were: (1) age from 7 to 20 years; (2) BMI > 95th percentile, according to CDC [46]. Exclusion criteria: we excluded children and adolescents affected by genetic/syndromic obesity, use of antihypertensive, antidiabetic, or lipid-lowering medication or medications known to cause lipodystrophy such as steroids that also affect body composition. On the same morning, the subjects underwent a medical interview, an anthropometric assessment (BMI, ABSI, WHtR, C-Index, and TMI), a measurement of systolic (SBP) and diastolic blood pressure (DPB), and blood sampling. The study was performed in accordance with the Declaration of Helsinki and the subjects gave their written informed consent. The study procedures were approved by the Ethical Committee of Milan University (report n. 23/2016).

### 4.2. Anthropometric Measurements

Weight and height were measured using a medical-certified scale and children’s medical-certified stadiometer, respectively following international guidelines [49]. BMI was calculated as [50]:

$$BMI=\frac{Weight\left(\mathrm{kg}\right)}{Height{\left(m\right)}^{2}}$$

BMI values were transformed into BMI z scores using CDC reference values [46]. Obesity was defined by BMI z score ≥ 1.645. WC was measured at the midpoint between the last rib and the iliac crest at the end of normal expiration, using an inextensible anthropometric tape positioned parallel to the floor [49].

### 4.3. Adiposity Index

ABSI was calculated using the following formula [51]:

$$ABSI=\frac{WC\left(m\right)}{BMI^{2/3}\times Height{\left(m\right)}^{1/2}}$$

ABSI values were transformed into ABSI *z*-score using NHANES values as reference [52].

WHtR was calculated as:

$$WHtR=\frac{WC\left(m\right)}{Height\left(m\right)}$$

C-Index was calculated using the following formula [53]:

$$C-Index=0.109^{-1}\times WC\times {\left(\frac{Weight\left(\mathrm{kg}\right)}{Height\left(m\right)}\right)}^{-1/2}$$

Finally, TMI was calculated using the following formula [31]:

$$TMI=\frac{Weight\left(\mathrm{kg}\right)}{Height{\left(m\right)}^{3}}$$

### 4.4. Clinical Assessment

A structured medical interview was carried out in order to obtain information about clinical history of the subject and possible drug therapies followed. Resting blood pressure (BP) was measured twice in sitting position after participants had rested for at least five minutes [54].

### 4.5. Laboratory Assessment

A blood sample was obtained in fasting state between 8:30 a.m. and 9:00 a.m. for measurement of plasma glucose, insulin, triglycerides (TG), HDL-cholesterol and analyzed in the same morning at the internal laboratory. The homeostasis model assessment of insulin resistance (HOMA-IR) was calculated using the following formula [55]:

$$HOMA-R=\frac{Glucose\left(\frac{\mathrm{mmol}}{L}\right)\times Insulin\left(\frac{\mathrm{mU}}{L}\right)}{22.5}$$

### 4.6. Metabolic Syndrome

Different criteria have been used for the diagnosis of metabolic syndrome (MetS) according to age groups.

For children aged from 7 to 10 years, metabolic syndrome was defined as reported by Ahrens et al. [56] in the IDEFICS study, with at least three of the following criteria: WC ≥ 90th percentile [57]; systolic or diastolic pressure ≥ 90th percentile [58]; triglycerides ≥ 90th percentile or HDL ≤ 10th percentile [59]; HOMA-IR ≥ 90th percentile or fasting blood sugar ≥ 90th percentile [60].

For children aged from 10 to 16 years, MetS was defined as proposed by IDF consensus [6], with WC ≥ 90th percentile for age and sex [61] plus at least 2 of the following criteria: fasting blood glucose ≥ 100 mg/dL (≥5–6 mmol/L); triglycerides ≥ 150 mg/dL (≥1.7 mmol/L); HDL < 40 mg/dL; SBP ≥ 130 mmHg or DBP ≥ 85 mmHg.

For patients age ≥ 16 years, MetS was defined following IDF criteria [62], with WC ≥ 94 cm for males and ≥80 cm for females, plus two of the following factors: fasting glucose > 100 mg/dL, TG ≥ 150 mg/dL, HDL-cholesterol < 40 mg/dL in males and <50 mg/dL in females, SBP ≥ 135 mmHg or DBP ≥ 85 mmHg.

### 4.7. Statistical Analysis

Several continuous variables did not follow a normal distribution and are therefore reported as 25th, 50th, and 75th percentile. Discrete variables are reported as frequency and percentage. Logistic regression models adjusted for sex and age were used to investigate the association between the anthropometric indices of interest (BMIz, ABSIz, TMI, C-Index, and WHtR) and the risk of MetS. We also investigated the joint contribution of each of the indices of interest and BMIz on the risk of MetS. However, because of evident collinearity problems, we could only investigate the joint contribution of BMIz and ABSIz. Multivariable fractional polynomials were used to ensure the linearity of continuous predictors with the outcomes. We used the Hosmer-Lemeshow test to assess the goodness of fit of the models. We reported McFadden pseudo-R2 as measures of predictive ability. To develop a relative comparison of the models we used the Akaike information criterion (AIC). The choice of the best predictive model was made on the basis of the lowest AIC value. Statistical analysis was performed using STATA version 12.0 (StataCorp, College Station, TX, USA).

## Figures and Tables

**Table 1:** Characteristics of the recruited subjects according to age class.

	7–9.9 Years *n* = 84			10–15.9 Years *n* = 229			16–19.9 Years *n* = 90			Total *n* = 403		
	P25	P50	P75	P25	P50	P75	P25	P50	P75	P25	P50	P75
Age (years)	8	9	9	12	13	14	17	17	19	10	13	16
Weight (kg)	43.0	48.5	53.8	63.5	74.0	85.3	87.3	93.5	104.4	56.6	74.8	90.3
Weight *z*-score	1.900	2.244	2.499	1.827	2.077	2.392	1.770	2.037	2.294	1.827	2.089	2.403
Height (cm)	133.1	139.3	143.6	151.2	157.4	165.4	163.3	169.9	175.7	145.5	156.7	167.5
Height *z*-score	0.240	0.948	1.803	−0.158	0.460	1.088	−0.414	0.051	0.546	−0.117	0.457	1.169
BMI (kg/m^2^)	23.7	24.8	27.1	27.0	29.1	31.4	30.5	32.6	36.1	26.6	29.4	32.3
BMI *z*-score	1.998	2.129	2.315	1.891	2.030	2.227	1.768	1.985	2.182	1.877	2.055	2.255
WC (cm)	78.0	81.5	86.2	86.0	92.5	98.8	96.6	101.8	109.4	84.8	92.0	100.7
ABSI	0.078	0.081	0.084	0.074	0.078	0.081	0.073	0.077	0.079	0.075	0.078	0.081
ABSI *z*-score	−0.029	0.705	1.214	−0.544	0.116	0.798	−0.395	0.389	0.849	−0.401	0.255	0.949
Total mass index	16.9	17.9	19.4	17.2	18.1	19.8	17.7	19.1	21.4	17.2	18.3	20.1
C-index	1.2	1.3	1.3	1.2	1.3	1.3	1.2	1.3	1.3	1.2	1.3	1.3
WHtR	0.56	0.59	0.62	0.55	0.58	0.62	0.56	0.60	0.65	0.56	0.59	0.62
Glucose (mg/dL)	79	83	89	82	88	93	84	90	96	81	87	93
Insulin	8.5	11.9	15.4	12.0	16.7	23.3	13.0	18.2	23.8	11.4	15.8	22.6
HOMA index	1.7	2.3	3.4	2.5	3.6	5.2	2.9	4.3	5.6	2.4	3.5	5.1
HDL (mg/dL)	41	48	54	39	47	55	42	49	54	40	47	55
TG (mg/dL)	61	79	109	64	84	121	58	85	117	61	83	119
SBP (mm Hg)	103	107	114	110	116	120	115	120	130	109	116	120
DBP (mm Hg)	56	60	66	60	65	70	70	70	80	60	66	71

Abbreviations: P25 = 25th percentile; P50 = 50th percentile; P75 = 75th percentile; WC = waist circumference; TG = triglycerides; SBP = systolic blood pressure; DBP = diastolic blood pressure.

**Table 2:** Distribution of metabolic syndrome and its components according to age class.

	7–10 Years		10–16 Years		16–20 Years		Total	
	N	%	N	%	N	%	N	%
Sex								
Female	55	65.5	122	53.3	47	52.2	224	55.6
Male	29	34.5	107	46.7	43	47.8	179	44.4
Total	84	100	229	100	90	100	403	100
High waist circumference								
No	0	0	47	20.5	6	6.7	53	13.2
Yes	84	100	182	79.5	84	93.3	350	86.8
Total	84	100	229	100	90	100	403	100
High glucose								
No	80	95.2	213	93	77	85.6	370	91.8
Yes	4	4.8	16	7	13	14.4	33	8.2
Total	84	100	229	100	90	100	403	100
High HOMA index								
No	35	41.7						
Yes	49	58.3						
Total	84	100						
High glucose or High HOMA								
No	35	41.7						
Yes	49	58.3						
Total	84	100						
High triglycerides								
No	53	63.1	198	86.5	76	84.4	327	81.1
Yes	31	36.9	31	13.5	14	15.6	76	18.9
Total	84	100	229	100	90	100	403	100
Low HDL								
No	66	78.6	170	74.2	59	65.6	295	73.2
Yes	18	21.4	59	25.8	31	34.4	108	26.8
Total	84	100	229	100	90	100	403	100
High triglycerides or Low HDL								
No	48	57.1						
Yes	36	42.9						
Total	84	100						
High systolic blood pressure								
No	65	77.4	203	88.6	62	68.9	330	81.9
Yes	19	22.6	26	11.4	28	31.1	73	18.1
Total	84	100	229	100	90	100	403	100
High diastolic blood pressure								
No	77	91.7	225	98.3	80	88.9	382	94.8
Yes	7	8.3	4	1.7	10	11.1	21	5.2
Total	84	100	229	100	90	100	403	100
Metabolic syndrome								
No	47	56	203	88.6	73	81.1	323	80.1
Yes	37	44	26	11.4	17	18.9	80	19.9
Total	84	100	229	100	90	100	403	100

**Table 3:** Association of anthropometric indexes with the risk of metabolic syndrome according to the age group.

	**Children < 10 Years**					**Children ≥ 10 Years**				
	BMIz	ABSIz	TMI	C-Index	WHR	BMIz	ABSIz	TMI	C-Index	WHR
Sex (Male)	−0.51	−0.17	−0.32	−0.20	−0.28	−0.11	−0.02	0.52	−0.21	0.23
	[−1.55, 0.52]	[−1.11, 0.77]	[−1.29, 0.65]	[−1.14, 0.75]	[−1.24, 0.67]	[−0.77, 0.55]	[−0.72, 0.68]	[−0.21, 1.26]	[−0.89, 0.48]	[−0.45, 0.92]
										
Age (years)	0.44	0.30	0.30	0.27	0.26	0.18 *	0.15 *	0.11	0.15 *	0.12
	[−0.16, 1.05]	[−0.36, 0.95]	[−0.30, 0.90]	[−0.39, 0.94]	[−0.37, 0.89]	[0.04, 0.31]	[0.02, 0.27]	[−0.01, 0.24]	[0.03, 0.28]	[−0.01, 0.25]
										
BMI *z*-score (CDC)	2.21 *					2.67 ***				
	[0.31, 4.12]					[1.44, 3.90]				
										
ABSI *z*-score		−0.10					0.37			
		[−0.58, 0.38]					[−0.01, 0.75]			
										
Total mass index			0.19					0.19 **		
			[−0.07, 0.46]					[0.07, 0.32]		
										
C-index				1.23					9.02 ***	
				[−5.61, 8.06]					[3.79, 14.25]	
										
Waist-to-Height ratio					5.39					12.32 ***
					[−4.64, 15.41]					[6.63, 18.01]
										
Constant	−8.82 *	−2.77	−6.37	−4.19	−5.68	−10.00 ***	−4.10 ***	−7.57 ***	−15.40 ***	−11.17 ***
	[−16.23, −1.41]	[−8.63, 3.08]	[−13.61, 0.87]	[−14.06, 5.67]	[−13.34, 1.98]	[−13.52, −6.47]	[−6.06, −2.14]	[−10.76, −4.37]	[−22.06, −8.74]	[−15.02, −7.31]
Observations	84	84	84	84	84	319	319	319	319	319
Pseudo R2	0.051	0.009	0.027	0.009	0.018	0.094	0.040	0.060	0.072	0.091
AIC	117	122	120	122	121	237	250	245	242	237
	**Female ≥ 10 Years**					**Male ≥ 10 Years**				
	BMIz	ABSIz	TMI	C-Index	WHR	BMIz	ABSIz	TMI	C-Index	WHR
Age (years)	0.13	0.11	0.07	0.11	0.06	0.20 *	0.19 *	0.22 *	0.21 *	0.21 *
	[−0.07, 0.32]	[−0.07, 0.29]	[−0.12, 0.26]	[−0.07, 0.29]	[−0.13, 0.25]	[0.01, 0.39]	[0.02, 0.37]	[0.04, 0.40]	[0.02, 0.39]	[0.03, 0.40]
										
BMI *z*-score (CDC)	1.24					3.84 ***				
	[−0.62, 3.10]					[1.99, 5.69]				
										
ABSI *z*-score		0.43					0.28			
		[−0.09, 0.95]					[−0.23, 0.79]			
										
Total mass index			0.09					0.35 ***		
			[−0.08, 0.26]					[0.17, 0.53]		
										
C-index				7.37 *					11.52 **	
				[0.70, 14.05]					[3.09, 19.96]	
										
Waist-to-Height ratio					7.94 *					18.79 ***
					[0.78, 15.10]					[9.87, 27.72]
										
Constant	−6.29 *	−3.61 **	−4.74 *	−12.67 **	−7.62 ***	−13.09 ***	−4.71 ***	−11.66 ***	−19.63 ***	−16.40 ***
	[−11.79, −0.78]	[−6.24, −0.97]	[−8.58, −0.89]	[−20.74, −4.61]	[−11.93, −3.31]	[−17.70, −8.48]	[−7.44, −1.98]	[−16.42, −6.90]	[−31.31, −7.96]	[−22.78, −10.02]
Observations	169	169	169	169	169	150	150	150	150	150
Pseudo R2	0.023	0.035	0.018	0.050	0.040	0.193	0.048	0.148	0.101	0.177
AIC	130	128	131	127	128	107	125	113	118	109

Values are regression coefficients and 95% confidence intervals [in brackets] obtained from logistic regression models adjusted for sex and age. Abbreviations: * *p* < 0.05 ** *p* < 0.01 *** *p* < 0.001.

**Table 4:** Joint contribution of body mass index (BMI) and body shape index (ABSI) in the prediction of metabolic syndrome.

	Children < 10 Years	Children ≥ 10 Years
Sex (Male)	−0.51	−0.38
	[−1.54, 0.52]	[−1.10, 0.34]
Age (years)	0.45	0.17 *
	[−0.16, 1.07]	[0.04, 0.31]
BMI *z*-score (CDC)	2.20 *	2.79 **
	[0.29, 4.12]	[1.56, 4.03]
ABSI *z*-score	−0.07	0.45 *
	[−0.57, 0.43]	[0.03, 0.86]
Constant	−8.82 *	−10.22 **
	[−16.29, −1.35]	[−13.58, −6.86]
Observations	84	319
Pseudo R2	0.051	0.115
AIC	119	233

Values are regression coefficients and 95% confidence intervals [in brackets] obtained from logistic regression models adjusted for sex and age. Abbreviations: * *p* < 0.05 ** *p* < 0.001.

## Supplementary Materials

Supplementary materials can be found at https://www.mdpi.com/1422-0067/21/11/4083/s1.
