# Supplementary material for: Evaluation of Different Adiposity Indices and Association with Metabolic Syndrome Risk in Obese Children: Is there a Winner?
Source: Int J Mol Sci. 2020 Jun 8;21(11):4083. doi: 10.3390/ijms21114083 (PMC7313019; doi:10.3390/ijms21114083)
Supplement: Supplementary file 1 [file ijms-21-04083-s001.pdf]

Table 1S: Association of anthropometric indexes with the risk of high blood pressure

	Children <10y					Children ≥10y				
	BMIz	ABSIz	TMI	C-Index	WHR	BMIz	ABSIz	TMI	C-Index	WHR
Sex (Male)	-0.03	0.39	0.22	0.36	0.26	0.16	0.68	0.79*	0.52	0.46
	[-1.19,1.13]	[-0.66,1.45]	[-0.89,1.33]	[-0.69,1.42]	[-0.83,1.35]	[-0.48,0.81]	[-0.04,1.39]	[0.13,1.45]	[-0.18,1.22]	[-0.15,1.07]
Age (years)	0.96*	0.71	0.73	0.69	0.66	0.29***	0.27***	0.23***	0.26***	0.25***
	[0.13,1.80]	[-0.09,1.51]	[-0.08,1.53]	[-0.11,1.49]	[-0.13,1.46]	[0.18,0.41]	[0.15,0.39]	[0.11,0.35]	[0.14,0.38]	[0.13,0.36]
BMI z-score (CDC)	3.34**					2.55***				
	[1.08,5.59]					[1.37,3.73]				
ABSI z-score		-0.19					-0.35			
		[-0.72,0.34]					[-0.75,0.05]			
Total mass index			0.27					0.19***		
			[-0.02,0.56]					[0.08,0.31]		
C-index				0.85					-1.44	
				[-6.46,8.16]					[-6.66,3.77]	
Waist-to-Height ratio					7.15					5.07
					[-4.12,18.43]					[-0.76,10.89]
Constant	-17.02**	-7.47*	-12.66*	-8.48	-11.40*	-11.30***	-5.79***	-9.03***	-3.81	-8.39***
	[-27.46,-6.58]	[-14.71,-0.24]	[-22.52,-2.80]	[-20.56,3.60]	[-21.85,-0.95]	[-14.33,-8.26]	[-7.85,-3.74]	[-11.76,-6.30]	[-9.89,2.27]	[-11.81,-4.97]
Observations	84	84	84	84	84	319	319	319	319	319
Pseudo R^2	0.124	0.042	0.073	0.038	0.054	0.138	0.090	0.109	0.077	0.087
AIC	93	101	98	101	99	269	283	277	287	284

	Female ≥10y					Male ≥10y				
	BMIz	ABSIz	TMI	C-Index	WHR	BMIz	ABSIz	TMI	C-Index	WHR
Age (years)	0.21**	0.14	0.06	0.14	0.11	0.40***	0.43***	0.40***	0.39***	0.39***
	[0.05,0.37]	[-0.01,0.29]	[-0.10,0.22]	[-0.01,0.29]	[-0.05,0.27]	[0.21,0.59]	[0.24,0.62]	[0.21,0.59]	[0.20,0.58]	[0.20,0.58]
BMI z-score (CDC)	3.08***					2.05**				
	[1.26,4.90]					[0.50,3.61]				
ABSI z-score		-0.15					-0.62*			
		[-0.70,0.41]					[-1.14,-0.09]			
Total mass index			0.22**					0.24**		
			[0.05,0.38]					[0.08,0.40]		
C-index				0.84					-4.07	
				[-6.41,8.09]					[-10.56,2.42]	
Waist-to-Height ratio					7.11					4.56
					[-1.06,15.28]					[-3.46,12.59]
Constant	-11.17***	-3.81**	-7.02***	-4.82	-7.53***	-11.65***	-7.44***	-11.84***	-1.99	-9.84***
	[-16.24,-6.10]	[-6.15,-1.47]	[-10.32,-3.72]	[-13.16,3.51]	[-12.01,-3.06]	[-15.56,-7.73]	[-10.44,-4.44]	[-15.97,-7.71]	[-10.72,6.74]	[-15.16,-4.53]
Observations	169	169	169	169	169	150	150	150	150	150
Pseudo R^2	0.099	0.025	0.067	0.023	0.046	0.186	0.182	0.187	0.151	0.149
AIC	137	147	141	148	144	133	133	132	138	138

Values are regression coefficients and 95% confidence intervals obtained from logistic regression models adjusted for sex and age

Abbreviations:

*p<0.05 **p<0.01 ***p<0.001

Table 1S: Association of anthropometric indexes with the risk of impaired fasting glucose

	Children <10y					Children ≥10y				
	BMIz	ABSIz	TMI	C-Index	WHR	BMIz	ABSIz	TMI	C-Index	WHR
Sex (Male)	-0.65	-0.46	-0.58	-0.43	-0.50	0.68	0.85	0.63	0.88	0.70
	[-2.69,1.40]	[-2.74,1.82]	[-2.81,1.65]	[-2.46,1.60]	[-2.43,1.42]	[-0.13,1.49]	[-0.02,1.72]	[-0.21,1.48]	[-0.00,1.77]	[-0.11,1.50]
Age (years)	0.04	0.00	-0.02	0.01	-0.03	0.21**	0.22**	0.22**	0.22**	0.23**
	[-0.74,0.82]	[-0.74,0.75]	[-0.75,0.70]	[-0.78,0.81]	[-0.78,0.73]	[0.06,0.37]	[0.07,0.37]	[0.08,0.37]	[0.06,0.37]	[0.08,0.37]
BMI z-score (CDC)	1.10					0.22				
	[-2.37,4.57]					[-1.18,1.62]				
ABSI z-score		-0.31					-0.22			
		[-1.44,0.82]					[-0.69,0.25]			
Total mass index			0.13					-0.04		
			[-0.32,0.57]					[-0.20,0.11]		
C-index				-2.67					-3.44	
				[-22.15,16.81]					[-9.78,2.91]	
Waist-to-Height ratio					1.18					-3.94
					[-25.98,28.35]					[-11.34,3.47]
Constant	-5.54	-2.72	-4.94	0.40	-3.32	-6.29**	-5.96***	-5.13*	-1.69	-3.71
	[-17.91,6.82]	[-9.26,3.83]	[-17.35,7.48]	[-23.22,24.01]	[-20.00,13.35]	[-10.53,-2.04]	[-8.46,-3.46]	[-9.18,-1.07]	[-9.99,6.62]	[-8.90,1.49]
Observations	84	84	84	84	84	319	319	319	319	319
Pseudo R^2	0.013	0.014	0.011	0.009	0.006	0.057	0.062	0.058	0.063	0.062
AIC	40	40	40	40	40	191	190	191	190	190

	Female ≥10y					Male ≥10y				
	BMIz	ABSIz	TMI	C-Index	WHR	BMIz	ABSIz	TMI	C-Index	WHR
Age (years)	0.15	0.15	0.16	0.15	0.17	0.26*	0.27**	0.26**	0.26*	0.27**
	[-0.10,0.39]	[-0.09,0.39]	[-0.06,0.38]	[-0.09,0.39]	[-0.05,0.39]	[0.06,0.46]	[0.07,0.47]	[0.07,0.46]	[0.06,0.46]	[0.07,0.47]
BMI z-score (CDC)	-0.25					0.35				
	[-2.46,1.95]					[-1.44,2.13]				
ABSI z-score		-0.22					-0.23			
		[-0.94,0.50]					[-0.86,0.39]			
Total mass index			-0.02					-0.04		
			[-0.24,0.20]					[-0.26,0.18]		
C-index				-2.66					-3.93	
				[-11.96,6.64]					[-12.48,4.62]	
Waist-to-Height ratio					-3.06					-4.09
					[-12.82,6.71]					[-14.82,6.63]
Constant	-4.29	-4.90**	-4.56	-1.65	-3.33	-6.57*	-5.90***	-5.18	-0.89	-3.54
	[-11.16,2.59]	[-8.55,-1.25]	[-10.12,0.99]	[-14.07,10.77]	[-10.50,3.84]	[-11.96,-1.17]	[-9.00,-2.79]	[-10.56,0.20]	[-12.48,10.70]	[-10.88,3.81]
Observations	169	169	169	169	169	150	150	150	150	150
Pseudo R^2	0.021	0.026	0.021	0.025	0.024	0.064	0.069	0.064	0.072	0.068
AIC	86	85	86	85	85	109	109	109	108	109

Values are regression coefficients and 95% confidence intervals obtained from logistic regression models adjusted for sex and age

Abbreviations:

*p<0.05 **p<0.01 ***p<0.001

Table 1S: Association of anthropometric indexes with the risk of high triglycerides

	Children <10y					Children ≥10y				
	BMIz	ABSIz	TMI	C-Index	WHR	BMIz	ABSIz	TMI	C-Index	WHR
Sex (Male)	0.07	0.05	0.12	0.01	0.06	0.18	-0.05	0.40	-0.16	0.29
	[-0.93,1.06]	[-0.92,1.01]	[-0.86,1.10]	[-0.96,0.98]	[-0.92,1.03]	[-0.46,0.82]	[-0.72,0.62]	[-0.28,1.07]	[-0.85,0.52]	[-0.36,0.94]
Age (years)	0.52	0.51	0.52	0.49	0.52	0.03	0.02	0.01	0.03	-0.01
	[-0.19,1.22]	[-0.22,1.24]	[-0.23,1.28]	[-0.23,1.20]	[-0.21,1.24]	[-0.11,0.16]	[-0.11,0.15]	[-0.13,0.14]	[-0.10,0.16]	[-0.14,0.13]
BMI z-score (CDC)	-0.06					1.17				
	[-2.16,2.05]					[-0.04,2.38]				
ABSI z-score		0.21					0.67***			
		[-0.29,0.71]					[0.29,1.04]			
Total mass index			-0.10					0.07		
			[-0.42,0.23]					[-0.05,0.19]		
C-index				2.91					10.61***	
				[-4.08,9.89]					[4.92,16.31]	
Waist-to-Height ratio					0.11					9.34**
					[-9.86,10.09]					[3.69,15.00]
Constant	-5.06	-5.25	-3.51	-8.62	-5.27	-4.70**	-2.28*	-3.47*	-15.52***	-7.51***
	[-12.53,2.40]	[-11.79,1.28]	[-11.15,4.14]	[-19.00,1.77]	[-13.00,2.46]	[-7.98,-1.41]	[-4.22,-0.34]	[-6.41,-0.53]	[-22.90,-8.14]	[-11.24,-3.77]
Observations	84	84	84	84	84	319	319	319	319	319
Pseudo R^2	0.020	0.026	0.024	0.026	0.020	0.018	0.056	0.008	0.070	0.043
AIC	116	116	116	116	116	263	253	265	249	256

	Female ≥10y					Male ≥10y				
	BMIz	ABSIz	TMI	C-Index	WHR	BMIz	ABSIz	TMI	C-Index	WHR
Age (years)	-0.05	-0.03	-0.08	-0.04	-0.11	0.09	0.08	0.09	0.09	0.09
	[-0.26,0.15]	[-0.23,0.17]	[-0.30,0.14]	[-0.24,0.16]	[-0.32,0.10]	[-0.09,0.26]	[-0.10,0.26]	[-0.08,0.27]	[-0.08,0.27]	[-0.08,0.27]
BMI z-score (CDC)	0.57					1.46				
	[-1.43,2.56]					[-0.05,2.97]				
ABSI z-score		0.81**					0.46			
		[0.28,1.34]					[-0.04,0.96]			
Total mass index			0.05					0.13		
			[-0.13,0.23]					[-0.04,0.30]		
C-index				11.75**					8.80*	
				[4.29,19.21]					[0.23,17.38]	
Waist-to-Height ratio					10.15*					9.69*
					[1.97,18.33]					[1.49,17.89]
Constant	-2.38	-1.64	-1.82	-16.02**	-6.51**	-6.01**	-3.05*	-5.43*	-14.33*	-8.84**
	[-7.57,2.80]	[-4.49,1.22]	[-5.49,1.84]	[-25.91,-6.14]	[-11.34,-1.67]	[-10.22,-1.79]	[-5.69,-0.41]	[-9.96,-0.90]	[-25.58,-3.08]	[-14.70,-2.97]
Observations	169	169	169	169	169	150	150	150	150	150
Pseudo R^2	0.006	0.088	0.005	0.098	0.051	0.034	0.032	0.024	0.048	0.049
AIC	132	122	132	120	126	133	134	135	132	131

Values are regression coefficients and 95% confidence intervals obtained from logistic regression models adjusted for sex and age

Abbreviations:

*p<0.05 **p<0.01 ***p<0.001

Table 1S: Association of anthropometric indexes with the risk of low HDL cholesterol

	Children <10y					Children ≥10y				
	BMIz	ABSIz	TMI	C-Index	WHR	BMIz	ABSIz	TMI	C-Index	WHR
Sex (Male)	-0.37	-0.07	-0.27	-0.15	-0.29	-0.22	-0.32	-0.19	-0.37	-0.21
	[-1.54,0.81]	[-1.18,1.04]	[-1.37,0.84]	[-1.26,0.96]	[-1.37,0.80]	[-0.70,0.27]	[-0.85,0.21]	[-0.73,0.35]	[-0.91,0.16]	[-0.71,0.28]
Age (years)	0.04	-0.10	-0.08	-0.15	-0.16	0.06	0.06	0.06	0.06	0.05
	[-0.67,0.74]	[-0.88,0.68]	[-0.79,0.62]	[-0.94,0.63]	[-0.91,0.59]	[-0.03,0.15]	[-0.03,0.15]	[-0.04,0.15]	[-0.03,0.15]	[-0.04,0.14]
BMI z-score (CDC)	1.95					0.12				
	[-0.70,4.59]					[-0.86,1.09]				
ABSI z-score		0.14					0.19			
		[-0.49,0.77]					[-0.09,0.47]			
Total mass index			0.24					0.01		
			[-0.09,0.57]					[-0.10,0.12]		
C-index				4.71					3.36	
				[-4.00,13.41]					[-0.62,7.33]	
Waist-to-Height ratio					9.96					2.94
					[-2.79,22.70]					[-1.90,7.77]
Constant	-5.76	-0.44	-4.85	-5.89	-5.74	-1.95	-1.69*	-1.90	-5.86*	-3.32*
	[-14.77,3.26]	[-7.39,6.52]	[-13.44,3.73]	[-18.12,6.35]	[-14.62,3.15]	[-4.37,0.47]	[-3.04,-0.33]	[-4.24,0.43]	[-10.96,-0.77]	[-6.31,-0.33]
Observations	84	84	84	84	84	319	319	319	319	319
Pseudo R^2	0.033	0.003	0.029	0.014	0.031	0.006	0.011	0.006	0.015	0.010
AIC	92	95	93	94	93	385	383	385	382	384

	Female ≥10y					Male ≥10y				
	BMIz	ABSIz	TMI	C-Index	WHR	BMIz	ABSIz	TMI	C-Index	WHR
Age (years)	0.07	0.10	0.13	0.09	0.09	0.02	0.03	0.03	0.03	0.02
	[-0.07,0.20]	[-0.04,0.23]	[-0.01,0.27]	[-0.04,0.22]	[-0.05,0.22]	[-0.11,0.15]	[-0.10,0.15]	[-0.10,0.15]	[-0.10,0.15]	[-0.10,0.15]
BMI z-score (CDC)	-1.41					1.45*				
	[-2.85,0.03]					[0.07,2.83]				
ABSI z-score		0.32					0.01			
		[-0.05,0.69]					[-0.42,0.45]			
Total mass index			-0.12					0.15		
			[-0.27,0.04]					[-0.01,0.30]		
C-index				3.71					2.85	
				[-1.22,8.65]					[-3.96,9.65]	
Waist-to-Height ratio					-0.03					6.62
					[-6.04,5.99]					[-1.11,14.36]
Constant	1.03	-2.19*	-0.38	-6.70*	-2.07	-4.38**	-1.42	-4.14*	-5.06	-5.35*
	[-2.65,4.70]	[-4.12,-0.26]	[-3.38,2.62]	[-13.24,-0.16]	[-5.79,1.65]	[-7.61,-1.14]	[-3.22,0.39]	[-7.37,-0.91]	[-13.75,3.63]	[-10.11,-0.60]
Observations	169	169	169	169	169	150	150	150	150	150
Pseudo R^2	0.026	0.025	0.021	0.021	0.009	0.028	0.001	0.021	0.006	0.021
AIC	208	208	209	209	211	173	178	174	177	174

Values are regression coefficients and 95% confidence intervals obtained from logistic regression models adjusted for sex and age

Abbreviations:

*p<0.05 **p<0.01 ***p<0.001
